# COVID-19 vaccine wastage in private and public healthcare facilities in KwaZulu-Natal, South Africa

**DOI:** 10.1093/inthealth/ihae056

**Published:** 2024-09-19

**Authors:** Viloshini Krishna Manickum, Lehlohonolo John Mathibe

**Affiliations:** Discipline of Pharmaceutical Sciences, School of Health Sciences, University of KwaZulu-Natal, 4041 Durban, South Africa; Discipline of Pharmaceutical Sciences, School of Health Sciences, University of KwaZulu-Natal, 4041 Durban, South Africa

**Keywords:** cold chain, COVID-19 vaccine wastage, public and private sector stock visibility systems, stock expiry, vaccine hesitancy, wastage minimization strategies

## Abstract

**Background:**

In KwaZulu-Natal (KZN), South Africa, COVID-19 vaccinations commenced in May 2021. This study investigated the extent and reasons for COVID-19 vaccine (C19V) wastage in KZN and strategies undertaken to mitigate loss.

**Methods:**

This two-phase multicenter study was conducted at private and public healthcare facilities from May 2021 to July 2022.

**Results:**

KZN reported 2% Pfizer and 1% Janssen C19V wasted, mainly due to expiry. C19V waste-minimization strategies reported by 100% public and private sector vaccination leads included cold chain monitoring, designated trained staff and the use of stock-management systems.

**Conclusions:**

The WHO’s risk-mitigation factors should be implemented continuously to minimize vaccine wastage.

## Introduction

KwaZulu-Natal (KZN), South Africa (SA), commenced the phased, unprecedented and urgent COVID-19 vaccination program (C19VP) during May 2021. Anecdotal evidence suggests that wastage was an inevitable consequence of the C19VP. This study investigates the extent and reasons for C19V wastage in KZN, SA, and the strategies undertaken to minimize wastage.

## Methods

This two-phase multicenter study was conducted at private and public healthcare facilities from May 2021 to July 2022. Phase 1 was retrospective, cross-sectional and observational, and comprised retrieving and analyzing Pfizer-BioNTech (Pfizer) and Janssen Biotech Inc. (Janssen) stock received, wasted and the reasons for wastage from vaccine distribution data and the Stock Visibility Systems (SVS) web portal, respectively. Phase 2 involved interviewing 12 and seven public and private sector COVID-19 vaccination leads, respectively, with collection, collation and analysis of data. The study proposal received full ethical approval from the relevant stakeholders.

## Results

Eighty-four out of 275 public, and 20 out of 39 private sector facilities, submitted Pfizer wastage data. Janssen wastage data were received from 28 out of 303 public and nine out of 42 private sector facilities. C19V suppliers’ data showed that KZN received, in total, 709 605 (i.e. 612 300 for the public plus 97 305 for the private sector) and 302 960 (i.e. 289 040 for the public plus 13 920 for the private sector) vials of Pfizer and Janssen vaccines, respectively. Altogether, about 2% (n=7210) and 1% (n=577) of the Pfizer and Janssen vaccines, respectively, were wasted.

Public and private sector facilities reported wastage of 1% (n=6516) and 0.7% (n=694) of Pfizer vials, respectively. Wastage of 0.1% (n=424) and 1% (n=153) of Janssen vials, respectively, was reported. As depicted in Table 1, the main reasons for public sector Pfizer wastage were expired stock (95%, n=6194), cold chain failure (2%, n=107) and low uptake at vaccination sites (0.4%, n=25). Cold chain failure (47%, n=72), damaged vials (28%, n=42) and low uptake at vaccination sites (18%, n=28) were the main reasons for private sector Janssen vaccine wastage.

**Table 1.** Reasons for vaccine wastage.

	Pfizer vaccine		Janssen vaccine	
Reasons	Vials lost in public sector	Vials lost in private sector	Vials lost in public sector	Vials lost in private sector
Cold chain failure	2% (n=107)	15% (n=104)	38% (n=159)	47% (n=72)
Contamination at vaccination site	0.02% (n=1)	0.1% (n=1)	0.2% (n=1)	0%
Damaged vials	0.3% (n=18)	4% (n=30)	3% (n=14)	28% (n=42)
Expired stock	95% (n=6194)	59% (n=411)	27% (n=115)	0%
Missing stock	0.1% (n=6)	0%	0.9% (n=4)	0.7% (n=1)
No reason(s) provided	2% (n=159)	10% (n=72)	25% (n=108)	0%
Reconstitution challenges	0%	0.9% (n=6)	0%	7% (n=10)
Theft during transportation	0.1% (n=5)	0%	3% (n=13)	0%
Vial label missing	0.02% (n=1)	0%	0%	0%
Wastage at vaccination site due to low demand	0.4% (n=25)	10% (n=70)	2% (n=10)	18% (n=28)

C19V waste-minimization strategies reported by 100% of public (N=12) and private sector (N=7) respondents included cold chain monitoring (CCM), designated trained staff managing C19V and the use of stock-management systems. The use of generators/inverters was reported by only 8% (n=1) public and 29% (n=2) of private sector respondents. Training on reconstitution of C19V was reported by only 8% (n=1) in the private sector. Public and private sector reported 25% (n=3) and 43% (n=3) using remaining reconstituted C19V for communities/administration staff on site, respectively. Only the private sector 14% (n=1) reported the use of social media for the marketing of C19VP.

## Discussion

The main findings of this study show that only 7787 vials of C19V were wasted in KZN, emphasizing under-reporting in KZN consistent with the findings of Lazarus et al.^1^ This was in contrast to the millions of vials lost reported by SA lay media.^2^ However, KZN, the second largest province in SA, has 73% rural districts^3^ with network connectivity challenges, which may have impacted on SVS and wastage reporting.

Although 100% CCM was reported in both sectors, and some public (8%) and private (29%) sector facilities reported the use of generators, higher rates of cold chain wastage were seen in the private sector. This highlights the need for more generators and intensified CCM in both sectors.

Stock-management systems were used by both sectors to monitor expiry dates and redistribute short-dated C19V. However, this had limited benefit as SA received short-dated vaccines and faced a lack of demand due to vaccine hesitancy, resulting in stockpiling of C19V^4^ and the expiry of a total of 6720 C19V.

Our study findings are similar to those from Australia, Hong Kong, the UK and other African countries reporting C19V wastages due to expiry and low vaccination uptake.^1^

Although 8% private sector leads reported reconstitution training, our study found that the private sector reported a higher percentage of damaged vials and of reconstitution challenges, emphasizing the need for advanced reconstitution and handling of vaccines training.

The private sector reported more wastage at vaccination sites, despite reporting more community engagement (43%) using remaining doses of vaccine daily and more marketing on social media than the public sector. Traditional and political leaders must be trained to address vaccine hesitancy and implement strategies for vaccine acceptance within KZN’s rural communities.^3^ Localized manufacturing may benefit SA by decreasing high unemployment rates^3^ with improved availability, accessibility and vaccine acceptancy.

## Conclusion

Although KZN reported low wastage rates, the WHO’s risk-mitigation factors and tools must be continuously implemented and monitored to minimize vaccine stock losses, thereby allowing evidence-based decision making. Furthermore, vaccine hesitancy may be decreased by community buy-in and information-sharing campaigns.

## Data Availability

The data supporting the findings of this study are available from the corresponding author upon reasonable request.
